# Effect of Oenological Additives on Oral Aroma Retention During Wine Tasting

**DOI:** 10.3390/foods15060975

**Published:** 2026-03-10

**Authors:** Rafael I. Velázquez-Martínez, Carolina Muñoz-González, Julia Crespo, María Ángeles Pozo-Bayón

**Affiliations:** 1 Instituto de Investigación en Ciencias de la Alimentación, CSIC-UAM, Nicolas Cabrera 9, 28049 Madrid, Spain; rafael.v.m@csic.es (R.I.V.-M.); c.munoz@csic.es (C.M.-G.); 2 Departamento de Investigación Agroalimentaria, Instituto Madrileño de Investigación y Desarrollo Rural, Agrario y Alimentario (IMIDRA), El Encín, A-2km 38.2, Alcalá de Henares, 28805 Madrid, Spain; julia.crespo.garcia@madrid.org

**Keywords:** winemaking practices, yeast mannoproteins, hydrolysable tannins, oral retention, aroma persistence, wine preference

## Abstract

The use of oenological additives is an emerging trend in winemaking aimed at improving technological properties. Recent studies suggest that these additives may also influence aroma persistence after wine consumption by modulating the retention of aroma compounds in the oral cavity. The aim of this study was to evaluate the effect of three commercial oenological additives, hydrolysable tannins (gallotannin and ellagitannin) and yeast mannoproteins, on the oral aroma retention of selected aroma compounds in red and white wines. Eight aromatised wines were prepared, including three red and three white wines with additives and two control wines without additives. Thirty-eight volunteers rinsed with each wine following the Spit-Off Odorant Measurement (SOOM) procedure. Oral aroma retention was calculated by comparing aroma levels in expectorated samples with those in wines prior to oral processing. Results showed that additive type significantly affected oral aroma retention (*p* < 0.05), depending on both the aroma compound and the wine matrix. In red wines, tannins increased the oral retention of most aroma compounds (5–20%), whereas in white wines, tannins reduced aroma retention. Mannoproteins enhanced oral aroma retention (5–40%) in both wine types. These results highlight the role of interactions between oenological additives, aroma compounds, and the wine matrix in modulating oral aroma retention.

## 1. Introduction

During wine tasting, aroma molecules are released into the buccal cavity, from where they migrate to the nasal cavity via the nasopharynx and interact with olfactory receptors through the retronasal pathway. Once activated, these receptors transmit sensory information to the brain for aroma identification. Two main forms of retronasal aroma perception can be distinguished during wine consumption: the immediate impression occurring just after swallowing, and the prolonged retronasal perception after swallowing, commonly referred to as aroma persistence, which is closely linked to wine preference [1].

Only aroma molecules that are adsorbed onto the oral and pharyngeal mucosa and are not metabolised by salivary enzymes can be perceived over an extended period [2,3]. The ability of these molecules to bind to the mucin protein layer forming the mucosal pellicle that covers the oropharyngeal cavity greatly determines their rate of release into the exhalation flows and, consequently, aroma persistence [1]. In addition to oral physiological factors (e.g., salivary flow and composition) [4,5,6], other parameters such as the type and physicochemical properties of aroma compounds (e.g., hydrophobicity and molecular structure), as well as the composition of the wine matrix (e.g., ethanol, polyphenols, polysaccharides), can influence the binding of aroma compounds to oral and pharyngeal mucosa [4,7,8,9,10].

Wine matrix composition depends on winemaking technology and can be modified through the use of oenological additives. Most of these products are derived from botanical sources such as grape skins, seeds, and stems, or from wine yeast. Their use has become an emerging trend in winemaking aimed at improving technological properties, including colloidal stabilisation, clarification, antioxidant capacity, and colour stability [11,12]. Among the most widely used oenological additives are oenotannins and yeast derivatives, such as mannoproteins.

Oenotannins are complex phenolic compounds found in grapes (*Vitis vinifera*) and other plant sources, including woods used in winemaking such as oak (*Quercus* spp.) and chestnut (*Castanea sativa*). Their chemical structure confers the ability to stabilise colloidal systems, promote clarification, and exert antioxidant activity while contributing to the optimisation of wine sensory properties [13,14,15,16,17,18,19]. Two main groups of tannins can be distinguished: hydrolysable and condensed tannins [20,21]. Hydrolysable tannins include ellagitannins and gallotannins, characterised by the presence of ellagic or gallic acid units esterified to a glucose core [22,23]. These structures, mainly derived from phenolic-rich woods, can undergo hydrolysis under acidic or enzymatic conditions, generating free phenolic acids that participate in wine stabilisation and modulation of organoleptic properties [21,24]. In contrast, condensed tannins, also known as proanthocyanidins, consist of oligomers and polymers of flavan-3-ols such as catechin and epicatechin linked by carbon–carbon bonds [20,22,25,26,27]. Unlike hydrolysable tannins, these compounds exhibit high resistance to hydrolytic degradation due to their polymeric nature [26].

Beyond their role in wine stabilisation, oenotannins influence key sensory attributes such as aroma, astringency, and bitterness [28,29,30,31,32,33]. The molecular mechanisms by which phenolic compounds interact with wine aroma molecules have been well established and recently reviewed [34,35]. For instance, phenolic compounds can bind non-polar aroma compounds through weak hydrophobic interactions [36], as well as polar volatile phenols via π–π stacking interactions involving aromatic rings [37,38,39]. Moreover, the capacity of wine phenolic compounds to modulate oral aroma retention and release has been confirmed using in vivo approaches [4,7,40].

Yeast derivatives, particularly mannoproteins, represent another important group of oenological additives with significant technological and sensory implications. These glycoproteins, mainly composed of mannose chains linked to a protein fraction, are structural components of the *Saccharomyces cerevisiae* cell wall and can be released during alcoholic fermentation [40,41,42,43,44,45]. Although mannoproteins are primarily applied to improve wine colloidal stability, they can also influence sensory perception by reducing tannin-related astringency and modulating the aromatic profile [43,46,47]. Their effect on aroma has been associated with interactions with volatile compounds through weak hydrophobic forces and/or through water adsorption in the surrounding medium, which affects aroma compound volatility [39,48,49,50,51,52].

In addition to their ability to interact with aroma compounds, both phenolic compounds and mannoproteins have been shown to interact with mucin, the main glycoprotein of the oral mucosa [10,53,54,55,56,57]. These findings support the hypothesis that oenological additives added to wines may favour the oral retention of certain aroma compounds. This hypothesis is consistent with recent sensory studies using time-intensity methodology, in which commercial oenological additives (hydrolysable tannins and mannoproteins), added at recommended winemaking dosages prior to bottling, were shown to differentially modify the persistence of fruity and woody aromas in white and red wines [58].

Quantifying oral aroma retention in vivo is analytically challenging, as it requires distinguishing between the initial concentration of aroma compounds in the product and the fraction that remains in the oral cavity after processing. The Spit-Off Odorant Measurement (SOOM) approach has been developed and successfully applied to address this challenge by enabling direct comparison between the concentration of target compounds in food or beverage matrices prior to oral exposure and that recovered in the corresponding expectorated samples. In this way, SOOM provides an analytical quantification of orally retained aroma compounds, allowing the investigation of matrix- and compound-dependent retention mechanisms under controlled conditions [4,7,59,60,61].

Therefore, the aim of the present study was to evaluate the effect of three commercial oenological additives—gallotannin, ellagitannin, and mannoprotein—on the oral retention of aroma compounds associated with fruity and woody wine nuances. The additives were incorporated into red and white wines at recommended winemaking dosages. Thirty-eight volunteers then performed the SOOM procedure, which quantifies oral aroma retention by comparing the concentration of target aroma compounds in wines before and after oral processing (i.e., in expectorated samples). Results were expressed as the percentage of aroma retained relative to its initial concentration in the wine. To our knowledge, this is the first study to directly relate the use of oenological additives to oral aroma retention, providing new insight into the mechanisms underlying prolonged retronasal aroma perception during wine tasting.

## 2. Materials and Methods

### 2.1. Wine Samples

Red and white wines produced from Tempranillo (red) and Malvar (white) grape varieties were industrially elaborated at the IMIDRA (Instituto Madrileño de Investiagción Agraria y Alimentaria) experimental winery (Alcalá de Henares, Madrid, Spain). These wines were used as control red (CRW) and control white wine (CWW). As previously reported [58], CRW presented a pH of 3.56, an alcohol content of 13.9% (*v*/*v*), 2.85 g/L tartaric acid, 0.03 g/L malic acid, 0.50 g/L glucose/fructose, and 56 mg/L total sulphur dioxide (SO_2_T). CWW showed a pH of 3.10, an alcohol content of 13.8% (*v*/*v*), 3.47 g/L tartaric acid, 0.53 g/L malic acid, 0.37 g/L glucose/fructose, and 26 mg/L SO_2_T.

From these control wines, three experimental wines were prepared by adding three different commercial oenological additives supplied by Laffort Ibérica S.A. (Guipúzcoa, Spain): a yeast-derived mannoprotein, a gallotannin, and an ellagitannin. For white wines, the additives were incorporated into CWW prior to bottling at concentrations of 1.4 mL/L (mannoprotein), 300 mg/L (gallotannin), and 700 mg/L (ellagitannin), according to the manufacturer’s recommendations. Mannoproteins were supplied in liquid form, whereas oenological tannins were provided as powders.

For red wines, the same additives and concentrations were applied, except for mannoproteins, which were added at 0.9 mL/L, in accordance with the manufacturer’s specifications and to match concentrations previously used in sensory studies conducted with the same wine types [58].

In total, eight wines were included in the study: four red wines: control (CRW), ellagitannin-added (ERW), gallotannin-added (GTRW), and mannoprotein-added (MRW); and four white wines: control (CWW), ellagitannin-added (EWW), gallotannin-added (GTWW), and mannoprotein-added (MWW).

The composition of the oenological additives and the characteristics of the wines are detailed in Table S1.

### 2.2. Aromatisation of Wines

The aroma profile of the wines was enhanced through the addition of selected volatile compounds chosen based on: (a) their distinct physicochemical properties (to better understand their behaviour during oral processing), and (b) their relevance to key sensory descriptors in white and red wines (i.e., fruity and woody attributes), as previously reported [62]. The compounds used, along with their main physicochemical characteristics, are listed in Table 1. All aroma compounds were of food grade and supplied by Merck (Darmstadt, Germany).

Stock solutions containing the aroma compounds corresponding to each aroma blend were prepared in food-grade ethanol. Wine aromatisation was performed 5–10 min prior to the in vivo assays. For this purpose, 15 mL of wine, previously poured into a wine glass, were spiked with 200 µL of the corresponding stock solution. Aromatised wines were immediately covered with Petri dishes to prevent aroma loss.

The final concentrations of the aroma compounds in the wines were as follows: 2,3-butanedione, 1400 µg/L; isoamyl acetate, 550 µg/L; ethyl acetate, 5000 µg/L; ethyl cinnamate, 12 µg/L; β-damascenone, 0.3 µg/L; trans- and cis-whiskylactone, 165 µg/L; vanillin, 55 µg/L; eugenol, 8 µg/L; guaiacol, 8 µg/L; and furaneol, 55 µg/L. These concentrations were selected based on previous sensory studies [58].

All compounds were added together to all wine samples (red and white), with and without oenological additives (control wines). As the aim of the present study was not to assess sensory perception but rather to evaluate the mechanistic behaviour of aroma compounds in the oral cavity, the experimental design focused on their retention capacity in the oral mucosa rather than on their individual sensory impact.

### 2.3. Spit-Off Odorant Measurement (SOOM) Procedure

**Figure 1:**
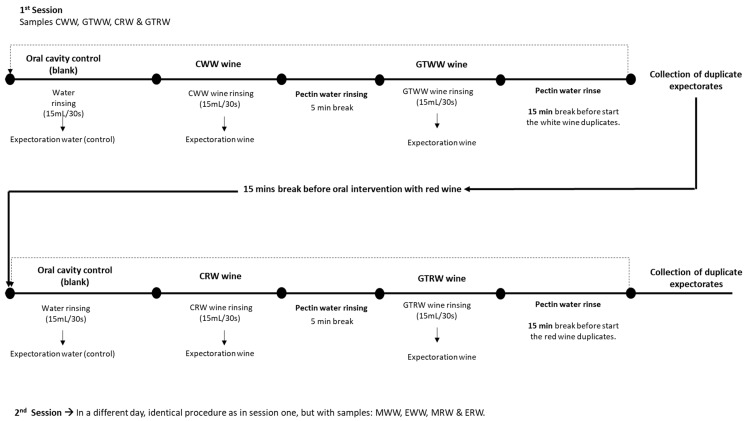
Schematic representation of the SOOM procedure. Dot lines means that the same procedure was applied for the duplicated samples.

Oral aroma retention was assessed using the SOOM method, as previously described [4,7,59,60,61]. This methodology allows quantification of aroma compounds retained in the oral cavity after wine rinsing by comparing their initial concentration in the wine with that recovered in the expectorated samples. A schematic representation of the experimental procedure is shown in Figure 1.

Thirty-eight healthy adults (24 women and 14 men; age range: 23–55 years) participated in the study, which was approved by the Bioethics Committee of the Spanish National Research Council (CSIC) (approval code: 008/2021). Exclusion criteria included smoking, pregnancy, and the presence of oral pathologies. All participants provided written informed consent and completed a food allergy questionnaire. Prior to the experimental sessions, participants received detailed instructions and performed preliminary trials to ensure familiarity with the procedure. Participants were instructed not to eat or drink anything except water for at least one hour before each session.

As shown in Figure 1, volunteers performed 30 s rinses with the wines, in duplicate, resulting in 16 expectorated wine samples per participant (four red and four white wines, each analysed in duplicate). Expectorated samples were collected over two separate sessions conducted on different days.

At the beginning of each session, participants rinsed their mouths with water for 30 s, and the expectorated water was collected in sterile 50 mL polypropylene Falcon tubes with screw caps (Biologix, Jinan, China) and stored at −80 °C. These samples were used as oral cavity blanks to verify the absence of the target volatile compounds prior to wine rinsing.

In the first session, after blank collection, participants rinsed with control white wine (CWW) for 30 s, and the expectorated wine was collected. Participants then rinsed their mouths with a pectin–water solution (500 mg/L) to remove potential polyphenol residues from the oral cavity [63]. After a 5 min break, the procedure was repeated using gallotannin-treated white wine (GTWW). Once completed, participants rinsed again with the pectin–water solution and rested for 15 min before repeating the same sequence with CWW and GTWW to obtain duplicate samples.

In the second session, conducted on a different day, the same protocol was applied to the remaining white wines (MWW and EWW) and to the red wines (MRW and ERW).

Immediately after collection of each expectorated wine or water sample, 0.5 g of CaCl_2_ (Panreac, Barcelona, Spain) was added to inactivate salivary enzymatic activity, as previously validated [59,63]. Samples were stored at −80 °C for approximately two weeks prior to aroma extraction.

In total, 16 aromatised wine samples (four red and four white wines, analysed in duplicate), 608 expectorated wine samples (38 participants × 4 white wines × 4 red wines × duplicate), and 76 expectorated water samples (oral cavity blanks from 38 participants, in duplicate) were subjected to aroma extraction and subsequent GC–MS analysis.

### 2.4. Analysis of Aroma Compounds

#### 2.4.1. Extraction

Aroma compounds from red and white wines (before oral processing) and from expectorated samples (water and red and white wines after oral processing) were extracted by liquid–liquid extraction using dichloromethane, as previously described [7,59,61,63].

Briefly, methyl nonanoate (100 µL of a 1 mg/L solution) was added as an internal standard to 10 mL of each sample (wine, water, or expectorated wine). Two consecutive extractions were then performed using 1 mL of dichloromethane (Scharlau, S.L., Barcelona, Spain) per extraction, with manual agitation repeated ten times. Samples were subsequently sonicated for 15 min in an ice bath and centrifuged at 5000× *g* for 15 min at 4 °C to separate the organic and aqueous phases.

The organic extracts were dried over anhydrous Na_2_SO_4_ (Panreac, Barcelona, Spain), filtered through glass wool, and spiked with 50 µL of 3-octanol (1 mg/mL), used as a second internal standard. The final volume was adjusted to 5 mL with dichloromethane, and the extracts were concentrated to 1.5 mL under a controlled nitrogen stream. All aroma extracts were stored at −80 °C until GC-MS analysis.

#### 2.4.2. Gas Chromatography–Mass Spectrometry (GC-MS)

Aroma extracts were analysed using a Bruker 436 gas chromatograph (Billerica, MA, USA) coupled to a Bruker EVOQ GC-TQ mass spectrometer. One µL of each extract was injected into the system in splitless mode with the injector temperature set at 250 °C.

The oven temperature programme consisted of an initial isothermal step at 40 °C for 5 min, followed by an increase to 136 °C at a rate of 4 °C/min, and a final increase to 250 °C at 6 °C/min. Helium was used as the carrier gas at a constant flow rate of 1 mL/min. Separation was achieved using a Rxi-5Sil MS capillary column (30 m × 0.25 mm i.d. × 0.25 µm film thickness; Restek, Bellefonte, PA, USA).

The MS transfer line was maintained at 250 °C and the ion source at 200 °C. Electron ionisation was performed at 70 eV, and ions were monitored in full-scan mode over a mass range of 35–350 m/z. A solvent delay of 5 min was used.

Identification of target volatile compounds was carried out by comparing retention times and mass spectra with those of reference standards analysed under identical chromatographic conditions, as well as with spectra from the NIST 2.0 mass spectral library. Peak identity was confirmed by matching both retention time and characteristic fragment ions. Chromatographic performance, including peak resolution and baseline stability, was routinely verified during data acquisition and processing.

Quantitative analysis was subsequently carried out in Selected Ion Monitoring (SIM) mode using characteristic ions selected from the reference standards. The following ions were monitored for quantification: ethyl cinnamate (m/z 131), β-damascenone (m/z 121), whiskylactone (m/z 99), vanillin (m/z 151), eugenol (m/z 164), guaiacol (m/z 109), furaneol (m/z 128), isoamyl acetate (m/z 70), and 2,3-butanedione (m/z 45). Methylnonanoate (m/z 74) and 3-octanol (m/z 83) were used as internal standards.

Quantitative comparisons among samples were performed using relative peak areas, calculated as the ratio between the absolute peak area of each compound and that of the internal standards. Chromatographic performance, including peak resolution and baseline stability, was routinely verified during data acquisition and processing.

### 2.5. Percentage of Aroma Retained in the Oral Cavity (%AR)

The percentage of aroma retained (%AR) in the oral cavity after rinsing with the different wine types was calculated (Equation (1)) by comparing the relative peak area of each aroma compound in the wine before oral processing $\left(A_{0}\right)$ with that measured in the corresponding expectorated sample after rinsing $\left(A_{exp}\right)$, as previously described [61]:

(1) $$\%AR=\left(\frac{A_{0}-A_{exp}}{A_{0}}\right)*100$$

where *A*_0_ represents the relative peak area of the aroma compound (normalised to the internal standard) in the wine prior to oral processing, and *A_exp_* corresponds to the relative peak area in the expectorated sample.

This calculation was performed for each red (CRW, ERW, GTRW, MRW) and white (CWW, EWW, GTWW, MWW) wines. The absence of endogenous target volatile compounds in the oral cavity was verified by analysing expectorated water samples collected as oral blanks under the same analytical conditions as the wine samples.

### 2.6. Statistical Analysis

Statistical analysis was performed using the Wilcoxon signed-rank test to evaluate differences in the percentage of aroma retained (%AR) between control wines and wines supplemented with oenological additives, considering the paired structure of the data (the same volunteers evaluated all wines). Normality of the data distribution was assessed prior to analysis.

Spearman rank correlation analysis was applied to assess the relationship between %AR and the physicochemical characteristics of the aroma compounds (boiling point, molecular weight, and log P).

Principal Component Analysis (PCA) was performed separately for red and white wines to explore multivariate relationships between oral aroma retention (%AR) and the physicochemical properties of the aroma compounds. The analysis included mean %AR values and the corresponding physicochemical parameters (boiling point, molecular weight, and log P). Variables were standardised prior to analysis, and components were extracted based on eigenvalues greater than 1. Score and loading plots were used to interpret the relationships among variables.

A significance level of 95% (*p* < 0.05) was applied in all statistical tests. All analyses were conducted using XLSTAT (version 2025.01).

## 3. Results

### 3.1. Identification of Target Aroma Compounds in Wines and Expectorated Samples

As described in the Materials and Methods section (Section 2.3), in order to increase the sensitivity of the SOOM-GC-MS approach used to assess oral aroma retention, red and white wines were spiked with ten volatile aroma compounds representative of different chemical classes associated with fruity and woody wine aroma descriptors [62].

Ethyl acetate and 2,3-butanedione were not detected in dichloromethane extracts either before oral processing or in expectorated samples (Table 1). Besides their relatively high polarity and volatility, limited recovery during dichloromethane-based liquid–liquid extraction and potential losses during nitrogen stream concentration may have contributed to their non-detection [64,65,66]. Early elution and possible overlap with the solvent front during GC-MS analysis may further compromise the detection of highly volatile compounds [65]. In the case of 2,3-butanedione, reversible binding with bisulfite in wine is well documented and may substantially reduce the free fraction available for extraction [67,68].

Furaneol and vanillin were detected only in red wines despite identical spiking levels in both matrices, suggesting matrix-dependent effects. The higher phenolic content and compositional complexity of red wines may influence aroma compound retention and partitioning behaviour through non-covalent interactions, thereby affecting extraction efficiency and apparent recovery [36,69,70].

As expected, none of the target aroma compounds were detected in the expectorated water samples used as oral cavity blanks (Table 1), confirming that the volatile compounds identified in the expectorated wine samples originated exclusively from the wine rinsing and not from endogenous oral sources.

### 3.2. Comparison of Oral Aroma Retention After Rinsing with Control Wines and Wines with Oenological Additives

#### 3.2.1. Red Wines

For red wines, nine out of the eleven volatile compounds added to the wines, eugenol, cis-whiskylactone, trans-whiskylactone, guaiacol, vanillin, furaneol, β-damascenone, ethyl cinnamate, and isoamyl acetate, were successfully recovered and identified both before and after oral processing (i.e., in the expectorated samples) (Table 1).

**Figure 2:**
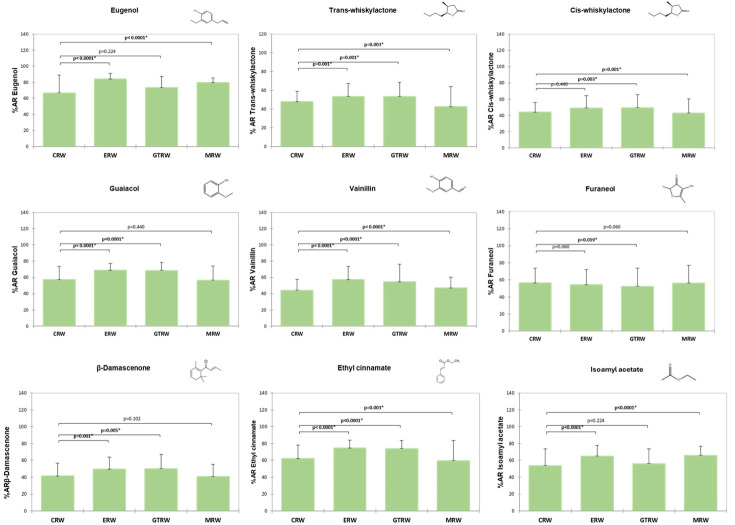
Percentage of aroma retained in the oral cavity of the volunteers (n = 38) and results from the Wilcoxon test to check significant differences between the control and treated wines. An asterisk (*) denotes significant differences (*p* < 0.05). CRW: red wine without additive (control); GTRW: red wine with gallotannin; MRW: red wine with mannoprotein; ERW: red wine with ellagitannin.

The comparison of the percentage of aroma retained in the oral cavity (%AR) for the control red wine (CRW, without additives) and the red wines supplemented with gallotannin (GTRW), ellagitannin (ERW), or mannoprotein (MRW) is shown in Figure 2 and detailed in Table S2. In the control wine, vanillin exhibited the lowest oral retention (approximately 34%), whereas volatile phenols (guaiacol and eugenol), furaneol, and the esters ethyl cinnamate and isoamyl acetate showed the highest %AR values (approximately 50–64%).

Figure 2 also shows a significant effect of oenological additives on oral aroma retention. Compared with the control wine, significant differences (*p* < 0.05) were observed for most tested aroma compounds. The magnitude of this effect depended both on the type of additive and on the aroma compound considered. Overall, tannins (gallotannin and ellagitannin) showed the strongest impact on oral aroma retention compared to mannoprotein.

In the gallotannin-supplemented wine (GTRW), oral aroma retention was significantly higher (*p* < 0.05) than in the control wine (CRW) for all compounds except eugenol and isoamyl acetate. Vanillin and ethyl cinnamate were the most affected compounds, exhibiting increases in oral retention greater than 20% compared to the control (Figure 2; Table S2).

A similar pattern was observed for the ellagitannin-supplemented wine (ERW). This additive significantly enhanced the oral retention of most target volatile compounds, with the exception of cis-whisky lactone. Again, vanillin and ethyl cinnamate showed the largest increases, exceeding 20% relative to the control wine.

In the mannoprotein-supplemented wine (MRW), the effect on oral aroma retention was compound-dependent. A significant increase (*p* < 0.05) was observed for most target compounds, except guaiacol, eugenol and β-damascenone. For most compounds, the magnitude of the increases was below 10% compared to the control, whereas isoamyl acetate, showed the largest enhancement (approximately 16%) (Figure 2; Table S2). In contrast, cis- and trans-whiskylactones and β-damascenone exhibited significantly lower oral retention in MRW than in CRW; however, these reductions were small, not exceeding 6%.

#### 3.2.2. White Wines

**Figure 3:**
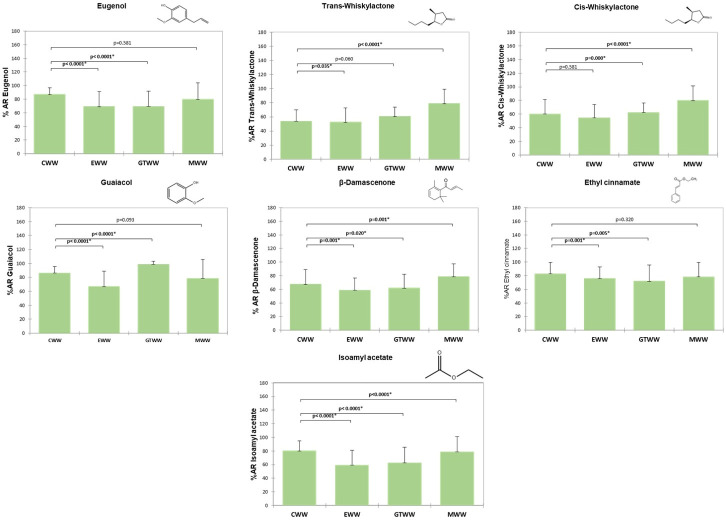
Percentage of aroma retained in the oral cavity of the volunteers (n = 38) and results from the Wilcoxon test to check significant differences between the control and treated wines. An asterisk (*) denotes significant differences (*p* < 0.05). CWW: white wine without additive (control); GTWW: white wine with gallotannin; MWW: white wine with mannoprotein; EWW: white wine with ellagitannin.

Figure 3 shows the comparison of the percentage of aroma retained in the oral cavity (%AR) for the control white wine (CWW) and the white wines supplemented with gallotannin (GTWW), ellagitannin (EWW), and mannoprotein (MWW). As previously indicated, some aroma compounds spiked into the wines were not detected in the aroma extracts obtained from the wines or the expectorated samples (Table 1). Therefore, Figure 3 presents the %AR values for the seven aroma compounds that were successfully identified in the samples: eugenol, cis- and trans-whiskylactones, guaiacol, β-damascenone, ethyl cinnamate, and isoamyl acetate.

In the control white wine (CWW), oral aroma retention ranged from approximately 50% for the least retained compounds (cis- and trans-whiskylactones) to more than 80% for the most retained compounds, namely the volatile phenols, guaiacol and eugenol, and the esters ethyl cinnamate and isoamyl acetate (Figure 3). This pattern is consistent with the high oral retention previously observed for these compounds in red wines (Figure 2).

As observed for red wines, the addition of tannins (gallotannin and ellagitannin) to white wines significantly affected the oral aroma retention of most target volatile compounds after rinsing (*p* < 0.05). Only cis-whiskylactone was not significantly affected by ellagitannin supplementation. Similarly, the addition of mannoproteins significantly influenced oral aroma retention for all aroma compounds, except eugenol and ethyl cinnamate.

Notably, the effect of tannins on aroma retention in white wines was opposite to that observed in red wines. With the exception of guaiacol, which showed an increase of approximately 13% in oral retention in the gallotannin-supplemented wine (GTWW) compared to the control, tannin addition generally resulted in a significant reduction in oral aroma retention relative to CWW (*p* < 0.05). This decrease affected a comparable number of compounds in both EWW and GTWW. The largest reduction (greater than 20%) was observed for isoamyl acetate in EWW, whereas trans- and cis-whiskylactones were the least affected compounds (<5%) in both tannin-supplemented wines (Figure 3; Table S2).

The white wine supplemented with mannoproteins (MWW) exhibited an opposite trend compared to tannin-added wines (Figure 3). Overall, mannoprotein addition resulted in a significant increase in oral aroma retention for most compounds (except eugenol and ethyl cinnamate). In particular, the oral retention of cis- and trans-whiskylactones, isoamyl acetate, and β-damascenone increased by approximately 4% for isoamyl acetate and up to 30–40% for the two whiskylactones. Only guaiacol showed a small but significant decrease (9%) in oral retention compared to the control.

### 3.3. Relationship Between Oral Aroma Retention and Physicochemical Aroma Characteristics

To gain insight into the relationship between oral aroma retention and the physicochemical properties of aroma compounds after rinsing with wines supplemented with different oenological additives, a principal component analysis (PCA) was performed separately for red and white wines. The analysis included both the percentage of aroma retained in the oral cavity (%AR) and the main physicochemical parameters of the aroma compounds—boiling point (BP), hydrophobicity (log P), and molecular weight (MW) (Table 1).

**Figure 4:**
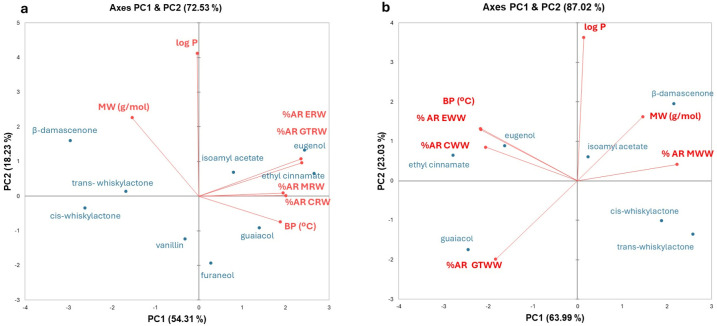
Biplot corresponding to the PCA showing the relationship among the percentage of aroma retained in the oral cavity (%AR), expressed as the average values of all 38 volunteers after rinsing with (**a**) red and (**b**) white wines, and the main physicochemical characteristics of the aroma molecules (MW: molecular weight; BP: boiling point; log P: hydrophobicity).

Figure 4 shows the PCA score and loading plots for red (Figure 4a) and white wines (Figure 4b). In red wines, the first two principal components explained more than 72% of the total variance (PC1: 54.3%; PC2: 18.2%). In white wines, the explained variance was higher, reaching 87% (PC1: 64.0%; PC2: 23.0%).

For red wines (Figure 4a), PC1 was strongly and positively associated with BP and negatively associated with MW, whereas log P was mainly related to PC2. All wine samples were closely grouped in the score plot, indicating a similar oral aroma retention behaviour across the four red wine types. Compounds such as ethyl cinnamate and the volatile phenols guaiacol and eugenol, characterised by relatively high BP values, were among the most retained aroma compounds across all red wines, including the control. A positive and significant correlation (0.7) between BP and percentage of aroma retention in tannin-added red wines (GTRW, ERW), was also found when applying Spearman correlation analysis (Table S3). However, BP alone did not fully explain the observed retention patterns, as vanillin, despite having the highest BP, did not cluster with these compounds and showed comparatively low %AR values in all wines.

An opposite trend was observed for MW, as compounds with lower molecular weights tended to show higher oral retention. In contrast, higher molecular weight compounds such as β-damascenone, vanillin, and the two whiskylactones, generally exhibited lower oral retention. Nevertheless, this relationship was not universal, since eugenol showed high oral retention despite its relatively high molecular weight (Table 1). Hydrophobicity (log P), which contributed mainly to PC2, showed a weaker influence on data variability; however, compounds with both high (ethyl cinnamate, isoamyl acetate) and low (eugenol, guaiacol) log P values were among the most retained aroma compounds in all red wines.

In the case of white wines (Figure 4b), the PCA revealed a stronger influence of wine type on oral aroma retention compared to red wines. PC1 was positively associated with MW and with %AR values in the mannoprotein-supplemented wine (MWW), and negatively associated with BP and %AR values in ellagitannin (EWW) and gallotannin-supplemented wines (GTWW). PC2 was strongly and positively related to log P. As shown in the score plot, high molecular weight compounds such as β-damascenone and isoamyl acetate exhibited the highest oral aroma retention after rinsing with MWW; these compounds also showed high hydrophobicity. In contrast, CWW and EWW clustered closely together, indicating a similar oral aroma retention behaviour that differed markedly from that observed for MWW. Ethyl cinnamate and eugenol, both characterised by relatively high BP values, were highly retained after rinsing with MWW, whereas guaiacol, the least hydrophobic compound among those tested, showed high oral retention in GTWW. A strong negative (−0.8) correlation between BP and oral aroma retention in MWW wines was also found after the application of Spearman correlation analysis (Table S3), while, on the contrary, a strong but positive correlation (>0.8) between BP and the percentage of oral aroma retention was found in CWW and EWW (Table S3), as also shown in the PCA (Figure 4b).

## 4. Discussion

The aim of the present study was to evaluate whether commercial oenological additives of different nature and composition, widely used to improve wine technological properties, are able to influence the interaction of aroma compounds within the oral cavity. This approach was motivated by previous findings showing that these additives can modify long-lasting aroma perception after wine consumption [58]. To address this objective, the Spit-Off Odorant Measurement (SOOM) methodology was applied, which allows the estimation of oral aroma retention by comparing the amount of aroma compounds present in the wine before and after oral processing (i.e., after wine rinsing).

This methodology assumes that aroma compounds not recovered in the expectorated wine samples are retained within the oral cavity through physical and/or chemical interactions with the oral mucosa [60,61,63]. Although aroma metabolism by salivary enzymes has been reported [3,9,59,71,72,73], this phenomenon is unlikely to play a major role under the experimental conditions of the present study. Wine acidity (pH ≈ 3.5) and ethanol content (~12%) are known to markedly reduce salivary enzymatic activity [73,74,75]. Enzymatic metabolism is expected to become relevant only after salivary pH recovery, which generally occurs several seconds after expectoration [76], and therefore is unlikely to happen in the experimental conditions of this study, that means, during mouth rinsing with the wine.

An important strength of this study is that the oenological additives were applied at concentrations recommended for industrial winemaking, providing technological relevance to the findings. Additive selection aimed to represent different product categories commonly used in oenology for different applications. Two hydrolysable tannins (gallotannin and ellagitannin), differing in structure and behaviour in wine, and a commercial yeast mannoprotein were selected. These additives were tested here in both red and white wines to investigate their behaviour toward aroma compounds under different wine matrix compositions.

To improve analytical sensitivity and better understand the behaviour of volatile compounds with diverse physicochemical properties, wines were aromatised with selected aroma molecules representative of fruity and woody notes. These compounds were added to both red and white wines regardless of aroma congruence, as the objective was not to perform a sensory evaluation but rather the study of physicochemical interactions occurring in the oral cavity. Sensory implications of similar systems have been addressed previously [58].

The study involved a relatively large number of volunteers (n = 38), allowing partial capture of inter-individual variability in oral physiology, such as differences in salivary composition, which has been identified as a relevant factor influencing oral aroma retention [5,10,77,78,79].

Regarding these findings, as shown in Table 1 and discussed in Section 3.1, not all aroma compounds added to the wines were recovered in the dichloromethane extracts. This can be attributed to the physicochemical heterogeneity of the selected aroma compounds, which influences their extraction efficiency, volatility, and behaviour during the analytical procedure, as well as to potential interactions with wine matrix components. Nevertheless, the majority of the target aroma compounds were consistently detected in both wines and expectorated samples. This provided a robust and chemically diverse dataset, allowing a representative evaluation of oral retention behaviour across compounds with different physicochemical properties (nine and seven aroma compounds in red and white wines, respectively).

Results also showed that in the control wine, oral aroma retention differed markedly among compounds. Esters (ethyl cinnamate and isoamyl acetate) and volatile phenols (eugenol and guaiacol) exhibited the highest retention values, consistent with previous reports showing relatively strong oral retention of hydrophobic esters [59], but also of polar volatile phenols despite their relatively low hydrophobicity [61]. This behaviour has been attributed to interactions between these volatile and phenolic compounds already present in the wine and adsorbed onto the oral mucosa [53]. Such interactions may involve weak hydrophobic forces with non-polar compounds (esters) and, in the case of polar compounds, π–π stacking interactions between the galloyl ring of phenolic compounds and the aromatic ring of volatile phenols, previously described in wine systems [36,37,38,39] and supported by in vivo and ex vivo studies [4,10].

The addition of oenological additives significantly modified oral aroma retention, with markedly different effects in red and white wines. This confirms the major role of the non-volatile wine matrix in modulating oral aroma retention and release, as previously reported in in vivo studies [7,10,63]. Additionally, this effect was different depending on the type of aroma compound. Although PCA (Figure 4), demonstrated that no single physicochemical aroma characteristic (hydrophobicity, molecular weight, or boiling point) could fully explain the observed oral retention patterns, highlighting the complexity of interactions between aroma compounds, wine macromolecules, and oral components.

Despite this, tannin-based additives increased the oral retention of most aroma compounds in red wines compared to the control, with increases ranging from approximately 5% to 24%. This effect was particularly pronounced for esters and volatile phenols, which are known to interact with phenolic compounds anchored to the oral mucosa [4,40]. Vanillin, the compound with the highest boiling point among those tested, also exhibited markedly enhanced oral retention in tannin-supplemented red wines relative to the control. In contrast, β-damascenone, a highly hydrophobic compound, showed lower retention than other less hydrophobic compounds such as ethyl cinnamate. This observation suggests that molecular structure and steric factors may limit interaction with phenolic constituents of the oral mucosa, as previously proposed [7].

PCA results indicated that all red wines clustered closely together, suggesting that endogenous polyphenols present in these red wines play a dominant role in oral aroma retention. The addition of exogenous tannins, although producing a significant effect on oral aroma retention compared to the control, was not sufficient to generate strong differentiation among the four red wine types.

In white wines, the oral retention behaviour of aroma compounds depending on the type of additive was very different, as shown in the PCA. Tannin-based additives (ellagitannin or gallotannin) have a more similar behaviour among them compared to mannoprotein.

Added tannins in white wines, generally reduced oral aroma retention compared to the control (around 9 and 20%) for most aroma molecules except for polar volatile phenols. These tannin-based additives, but also the endogenous presence of other non-flavonoid phenolic compounds present in these wines (phenolic acids) [80] could bind to the oral mucosa and trigger interactions with polar volatile phenols, as evidenced in white wines [7]. However, the amount of added tannins was possibly not enough to bind to the oral epithelium and trigger hydrophobic interactions with non-polar aroma compounds.

Additionally, these added tannins can interact with small phenol-binding proteins from circulating saliva (such as PRPs and cystatins) [81], leading to the formation of large aggregates that can incorporate aroma molecules [76,79,80], reducing their availability for mucosal retention. This hypothesis is supported by recent findings showing increased release of phenol-binding salivary proteins upon tannin addition at the same dosage and wine type [81]. However, further studies are needed to elucidate the underlying molecular mechanisms of these interactions in the presence of different types of phenolic compounds.

Regarding the effect of mannoproteins, the present results show that these additives can increase oral aroma retention in both red and white wines, although the magnitude and direction of the effect strongly depended on the aroma compound and the wine matrix. Mannoproteins are known to interact with aroma compounds through weak hydrophobic interactions and water structuring effects, as extensively reported in model wine systems [48,49,50,51,52], and they are also able to interact with mucin, the main glycoprotein of the oral mucosal pellicle [55,56]. These properties suggest that mannoproteins may favour aroma retention in the oral cavity through indirect interactions involving both wine and oral macromolecules.

In red wines, mannoproteins moderately increased the oral retention of hydrophobic esters, in agreement with previous studies describing aroma–mannoprotein interactions in model wines [48,49,50,51,52]. The differences observed among esters may be partly attributed to variations in hydrophobicity and molecular structure, which could generate steric hindrance effects that limit their interactions with mannoproteins [48,49] or with oral mucosal glycoproteins [7]. Despite its polar nature, eugenol also showed enhanced oral retention in red wines supplemented with mannoproteins. This behaviour may be explained by the formation of polyphenol-mannoprotein complexes that become anchored to the oral mucosa, subsequently favouring the retention of polar aroma compounds through π–π stacking, as previously explained. The fact that in white wines this enhancing oral retention effect was not observed for these polar volatile compounds, agrees with this explanation.

Lactones exhibited, however, a reduced oral retention in red wines containing mannoproteins, or even, as in the case of β-damascenone, were not affected by this additive. One plausible hypothesis is that, in red wines, mannoproteins preferentially interact with endogenous polyphenols, which are abundant in this matrix. These interactions may limit the ability of mannoproteins to bind directly to oral mucin, thereby reducing their potential to promote lactone retention.

Conversely, in white wines, mannoproteins markedly enhanced the oral retention of lactones (trans- and cis-whisky lactones), with increases of up to 30–40%. This behaviour is consistent with the strong interactions (Schiff covalent interactions) previously reported between lactones and salivary proteins using in vitro systems [76,78], as well as with the slow oral release kinetics of lactones observed in in vivo oral monitoring studies [40]. In white wines, which contain much lower levels of flavonoid-type polyphenols, mannoproteins may remain more available to interact with oral mucin, thereby reinforcing lactone–mucin interactions and promoting lactone retention in the oral cavity.

Overall, the present study demonstrates that oenological additives can significantly modulate oral aroma retention, with effects strongly dependent on wine matrix composition and aroma compound type. The wide structural diversity of commercial oenological additives suggests that targeted selection of these additives could offer new opportunities to modulate retronasal aroma release and aroma persistence. However, additional studies using more controlled systems (synthetic wines) using in vitro oral models will be required to fully elucidate the molecular mechanisms involved in these interactions to optimise the use of these additives to improve wine aroma characteristics during wine tasting beyond their traditional technological roles.

In spite of the novelty of these findings, several limitations of the present study should be acknowledged. The experiments were conducted using aromatised wines, and potential differences between externally added and naturally occurring aroma compounds were not evaluated. Although aroma–oral mucosa interactions are primarily governed by physicochemical properties and are expected to be similar regardless of compound origin, possible differences in interaction behaviour cannot be excluded. In spite of the obvious differences in phenolic composition between white and red wines, which has been described in the literature, the exact role of these compounds in the aroma-oenological additive–oral mucosa interactions should be assessed in future and more dedicated studies using specific phenolic compounds. Finally, the sensory relevance of the observed oral retention effect was not directly assessed, and it is important to assess that oral retention is discussed as a potential contributing mechanism rather than as a direct measurement of sensory persistence. Additionally, the study was limited to Tempranillo and Malvar red and white wines, which may restrict the generalisation of the findings to other wine types.

## 5. Conclusions

This study demonstrates that oenological additives, specifically hydrolysable tannins and yeast mannoproteins, applied at typical winemaking concentrations, can significantly influence the oral retention of wine aroma compounds. The magnitude and direction of these effects differed between red and white wines and depended on the chemical nature of the aroma compounds.

In red wines, both gallotannin and ellagitannin increased the oral retention of most tested aroma compounds (approximately 5–20%) compared to the control wine. Vanillin, ethyl cinnamate, and isoamyl acetate were among the compounds showing the highest oral retention. Additionally, these tannin-based additives enhanced the retention of relatively polar compounds such as eugenol and guaiacol. In contrast, tannin addition reduced oral aroma retention in white wines. Yeast mannoproteins enhanced oral aroma retention for most compounds in both wine types, with particularly pronounced effects for lactones in white wines, where increases of up to 30–40% were observed.

The observed differences, especially in white wines, appear to be linked to the wine matrix composition, which may modulate interactions between aroma compounds, the oral mucosa, and added macromolecules such as tannins and mannoproteins. These results highlight the complexity of the molecular interactions occurring in the oral cavity between aroma compounds and both endogenous and exogenous wine components.

## Figures and Tables

**Table 1:** Detection status of target volatile compounds added to red and white wines before and after oral processing, as determined by dichloromethane extraction followed by GC–MS analysis.

Targeted Compounds	Physicochemical Characteristics			Before Oral Processing		After Oral Processing								
	Log P	MW	BP	RW	WW	OCB	CRW	GTRW	ERW	MRW	CWW	GTWW	EWW	MWW
2,3-Butanedione (F)	−1.34	86	88	n.d.	n.d.	n.d.	n.d.	n.d.	n.d.	n.d.	n.d.	n.d.	n.d.	n.d.
Isoamyl acetate (F)	2.26	130	142	d.	d.	n.d.	d.	d	d.	d.	d.	d.	d.	d.
Ethyl acetate (F)	0.68	88	77	n.d.	n.d.	n.d.	n.d.	n.d.	n.d.	n.d.	n.d.	n.d.	n.d.	n.d.
Ethyl cinnamate (F)	2.99	176	271	d.	d.	n.d.	d.	d.	d.	d.	d.	d.	d.	d.
β-Damascenone (F)	3.20	190	110	d.	d.	n.d.	d.	d.	d.	d.	d.	d.	d.	d.
trans- and cis- Whisky lactones (W)	1.81	156	142	d.	d	n.d.	d.	d.	d.	d.	d.	d.	d.	d.
Vanillin (W)	1.20	152	285	d.	n.d.	n.d.	d.	d.	d.	d.	n.d.	n.d.	n.d.	n.d.
Eugenol (W)	1.83	164	254	d.	d.	n.d.	d.	d.	d.	d.	d	d.	d.	d.
Guaiacol (W)	1.34	124	205	d.	d.	n.d.	d.	d.	d.	d	d.	d.	d.	d.
Furaneol (W)	0.95	128	193	d.	n.d.	n.d.	d.	d.	d.	d.	n.d.	n.d.	n.d.	n.d.

Log P: hydrophobicity; MW: Molecular Weight; BP: Boiling Point (°C). RW: Red wine; WW: White wine; OCB: Oral cavity blank (expectorated water); CRW: red wine without additive (control); GTRW: red wine with gallotannin; ERW: red wine with ellagitannin; MRW: red wine with mannoprotein; CWW: white wine without additive (control); GTWW: white wine with gallotannin; EWW: white wine with ellagitannin; MWW: white wine with mannoprotein. d.: detected; n.d.: non-detected compound. Compounds related to the (F) fruity and (W) woody aroma descriptors [62].

## Data Availability

The original contributions presented in this study are included in the article/Supplementary Material. Further inquiries can be directed to the corresponding author.

## Supplementary Materials

The following supporting information can be downloaded at: https://www.mdpi.com/article/10.3390/foods15060975/s1, Table S1: Oenological additives and wines prepared for the present study; Table S2: Percentages of aroma retained in the oral cavity of the volunteers (n = 38) after rinsing with the different types of red and white wines used in this study; Table S3: Spearman Correlation analysis between percentage of oral aroma retention and physicochemical aroma characteristics for the different wines.

## References

[1] Muñoz-González C., Pozo-Bayón M.Á., Canon F. (2021). Understanding the Molecular Basis of Aroma Persistence Using Real-Time Mass Spectrometry. ACS Symposium Series.

[2] Pozo-Bayón M.A., Muñoz-González C. (2022). Oral Processing of Wine. Oral Processing and Consumer Perception.

[3] Muñoz-González C., Brule M., Martin C., Feron G., Canon F. (2022). Molecular mechanisms of aroma persistence: From noncovalent interactions between aroma compounds and the oral mucosa to metabolization of aroma compounds by saliva and oral cells. Food Chem..

[4] Perez-Jiménez M., Chaya C., Pozo-Bayón M.Á. (2019). Individual differences and effect of phenolic compounds in the immediate and prolonged in-mouth aroma release and retronasal aroma intensity during wine tasting. Food Chem..

[5] Muñoz-González C., Canon F., Feron G., Guichard E., Pozo-Bayón M.A. (2019). Assessment wine aroma persistence by using an in vivo PTR-TOF-MS approach and its relationship with salivary parameters. Molecules.

[6] Criado C., Chaya C., Fernández-Ruíz V., Álvarez M.D., Herranz B., Pozo-Bayón M.Á. (2019). Effect of saliva composition and flow on inter-individual differences in the temporal perception of retronasal aroma during wine tasting. Food Res. Int..

[7] Esteban-Fernández A., Muñoz-González C., Jiménez-Girón A., Pérez-Jiménez M., Pozo-Bayón M.Á. (2018). Aroma release in the oral cavity after wine intake is influenced by wine matrix composition. Food Chem..

[8] Muñoz-González C., Pérez-Jiménez M., Pozo-Bayón M.Á. (2020). Oral persistence of esters is affected by wine matrix composition. Food Res. Int..

[9] Muñoz-González C., Pérez-Jiménez M., Criado C., Pozo-Bayón M.Á. (2019). Effects of ethanol concentration on oral aroma release after wine consumption. Molecules.

[10] Perez-Jiménez M., Esteban-Fernández A., Muñoz-González C., Pozo-Bayón M.A. (2020). Interactions among odorants, phenolic compounds, and oral components and their effects on wine aroma volatility. Molecules.

[11] Kolling J., Futigami L.S., Assumpção T.I., Mazon-Freitas L., Arcari S.G., Burin V.M. (2025). The role of enological additives in the pre-fermentation stage: Influence on browning index and phenolic composition of Goethe grape must and wine. Eur. Food Res. Technol..

[12] Li S., Zhai H., Ma W., Duan C., Yi L. (2023). Yeast mannoproteins: Organoleptic modulating functions, mechanisms, and product development trends in winemaking. Food Front..

[13] Liao H., Cai Y., Haslam E. (1992). Polyphenol interactions. Anthocyanins: Copigmentation and colour changes in red wines. J. Sci. Food Agric..

[14] Castañeda-Ovando A., Pacheco-Hernández M.d.L., Páez-Hernández M.E., Rodríguez J.A., Galán-Vidal C.A. (2009). Chemical studies of anthocyanins: A review. Food Chem..

[15] Neves A.C., Spranger M.I., Zhao Y., Leandro M.C., Sun B. (2010). Effect of addition of commercial grape seed tannins on phenolic composition, chromatic characteristics, and antioxidant activity of red wine. J. Agric. Food Chem..

[16] Canuti V., Puccioni S., Giovani G., Salmi M., Rosi I., Bertuccioli M. (2012). Effect of Oenotannin Addition on the Composition of Sangiovese Wines from Grapes with Different Characteristics. Am. J. Enol. Vitic..

[17] Vignault A., González-Centeno M.R., Pascual O., Gombau J., Jourdes M., Moine V., Iturmendi N., Canals J.M., Zamora F., Teissedre P.L. (2018). Chemical characterization, antioxidant properties and oxygen consumption rate of 36 commercial oenological tannins in a model wine solution. Food Chem..

[18] Alcalde-Eon C., Pérez-Mestre C., Ferreras-Charro R., Rivero F.J., Heredia F.J., Escribano-Bailón M.T. (2019). Addition of Mannoproteins and/or Seeds during Winemaking and Their Effects on Pigment Composition and Color Stability. J. Agric. Food Chem..

[19] Ben Aziz M., Moutaoikil M., Zeng L., Mouhaddach A., Boudboud A., Hajji L., Hajjaj H. (2024). Review on oenological tannins: Conventional and emergent extraction techniques, and characterization. J. Food Meas. Charact..

[20] Paissoni M.A., Bitelli G., Vilanova M., Montanini C., Río Segade S., Rolle L., Giacosa S. (2022). Relative impact of oenological tannins in model solutions and red wine according to phenolic, antioxidant, and sensory traits. Food Res. Int..

[21] Maioli F., Sanarica L., Cecchi L., Zanoni B., Mulinacci N., Canuti V. (2023). Characterization of 20 Oenological Tannins from Different Botanical Origins for Formulation of Blends with Redox Potential Tuning Ability in Model Wine Solution. Antioxidants.

[22] Hagerman A.E. (2011). Extraction of phenolics from plants, Sephadex LH 20 and Separation of tannin from non-tannin phenolics. The Tannin Handbook.

[23] Michel J., Jourdes M., Le Floch A., Giordanengo T., Mourey N., Teissedre P.L. (2013). Influence of wood barrels classified by NIRS on the ellagitannin content/composition and on the organoleptic properties of wine. J. Agric. Food Chem..

[24] Canuti V., Cantu A., Picchi M., Lerno L.A., Tanabe C.K., Zanoni B., Heymann H., Ebeler S.E. (2020). Evaluation of the intrinsic and perceived quality of sangiovese wines from California and Italy. Foods.

[25] Ma W., Guo A., Zhang Y., Wang H., Liu Y., Li H. (2014). A review on astringency and bitterness perception of tannins in wine. Trends Food Sci. Technol..

[26] Schofield P., Mbugua D.M., Pell A.N. (2001). Analysis of condensed tannins: A review. Anim. Feed Sci. Technol..

[27] Versari A., Du Toit W., Parpinello G.P. (2013). Oenological tannins: A review. Aust. J. Grape Wine Res..

[28] Bautista-Ortín A.B., Martínez-Cutillas A., Ros-García J.M., López-Roca J.M., Gómez-Plaza E. (2005). Improving colour extraction and stability in red wines: The use of maceration enzymes and enological tannins. Int. J. Food Sci. Technol..

[29] Harbertson J.F., Parpinello G.P., Heymann H., Downey M.O. (2012). Impact of exogenous tannin additions on wine chemistry and wine sensory character. Food Chem..

[30] Larcher R., Tonidandel L., Román Villegas T., Nardin T., Fedrizzi B., Nicolini G. (2015). Pre-fermentation addition of grape tannin increases the varietal thiols content in wine. Food Chem..

[31] Chen K., Escott C., Loira I., Del Fresno J.M., Morata A., Tesfaye W., Calderon F., Benito S., Suárez-Lepe J.A. (2016). The effects of pre-fermentative addition of oenological tannins on wine components and sensorial qualities of red wine. Molecules.

[32] Li L., Li Z., Wei Z., Yu W., Cui Y. (2020). Effect of tannin addition on chromatic characteristics, sensory qualities and antioxidant activities of red wines. RSC Adv..

[33] Corona O., Bambina P., De Filippi D., Cinquanta L. (2021). Influence of pre-fermentative addition of aqueous solution tannins extracted from oak wood (*Quercus petraea*) on the composition of Grillo wines. Eur. Food Res. Technol..

[34] Pittari E., Moio L., Piombino P. (2021). Interactions between Polyphenols and Volatile Compounds in Wine: A Literature Review on Physicochemical and Sensory Insights. Appl. Sci..

[35] Pittari E., Piombino P., Andriot I., Cheynier V., Cordelle S., Feron G., Gourrat K., Le Quéré J.L., Meudec E., Moio L. (2022). Effects of oenological tannins on aroma release and perception of oxidized and non-oxidized red wine: A dynamic real-time in-vivo study coupling sensory evaluation and analytical chemistry. Food Chem..

[36] Dufour C., Bayonove C.L. (1999). Interactions between wine polyphenols and aroma substances. An insight at the molecular level. J. Agric. Food Chem..

[37] Aronson J., Ebeler S.E. (2004). Effect of Polyphenol Compounds on the Headspace Volatility of Flavors. Am. J. Enol. Vitic..

[38] Jung D., Ebeler S. (2003). Headspace solid-phase microextraction method for the study of the volatility of selected flavor compounds. J. Agric. Food Chem..

[39] Rodríguez-Bencomo J.J., Muñoz-González C., Andújar-Ortiz I., Martín-Álvarez P.J., Moreno-Arribas M.V., Pozo-Bayón M.Á. (2011). Assessment of the effect of the non-volatile wine matrix on the volatility of typical wine aroma compounds by headspace solid phase microextraction/gas chromatography analysis. J. Sci. Food Agric..

[40] Muñoz-González C., Criado C., Pérez-Jiménez M., Pozo-Bayón M.Á. (2021). Evaluation of the effect of a grape seed tannin extract on wine ester release and perception using in vitro and in vivo instrumental and sensory approaches. Foods.

[41] Pozo-Bayón M.Á., Andújar-Ortiz I., Moreno-Arribas M.V. (2009). Scientific evidences beyond the application of inactive dry yeast preparations in winemaking. Food Res. Int..

[42] Comuzzo P., Tat L., Fenzi D., Brotto L., Battistutta F., Zironi R. (2011). Interactions between yeast autolysates and volatile compounds in wine and model solution. Food Chem..

[43] Del Barrio-Galán R., Pérez-Magariño S., Ortega-Heras M. (2011). Techniques for improving or replacing ageing on lees of oak aged red wines: The effects on polysaccharides and the phenolic composition. Food Chem..

[44] Del Barrio-Galán R., Ortega-Heras M., Sánchez-Iglesias M., Pérez-Magariño S. (2012). Interactions of phenolic and volatile compounds with yeast lees, commercial yeast derivatives and non toasted chips in model solutions and young red wines. Eur. Food Res. Technol..

[45] Del Barrio-Galán R., Medel-Marabolí M., Peña-Neira Á. (2015). Effect of different aging techniques on the polysaccharide and phenolic composition and sensory characteristics of Syrah red wines fermented using different yeast strains. Food Chem..

[46] Rinaldi A., Blaiotta G., Aponte M., Moio L. (2016). Effect of yeast strain and some nutritional factors on tannin composition and potential astringency of model wines. Food Microbiol..

[47] Pérez-Magariño S., Martínez-Lapuente L., Bueno-Herrera M., Ortega-Heras M., Guadalupe Z., Ayestarán B. (2015). Use of Commercial Dry Yeast Products Rich in Mannoproteins for White and Rosé Sparkling Wine Elaboration. J. Agric. Food Chem..

[48] Lubbers S., Voilley A., Feuillat M., Charpentier C. (1994). Influence of mannoproteins on aroma compound behavior in a model wine. LWT-Food Sci. Technol..

[49] Lubbers S., Charpentier C., Feuillat M., Voilley A. (1994). Influence of Yeast Walls on the Behavior of Aroma Compounds in a Model Wine. Am. J. Enol. Vitic..

[50] Dong H., Guo Z., Ma Y., Lin J., Zhai H., Ren D., Li S., Yi L. (2024). Organoleptic modulation functions and physiochemical characteristics of mannoproteins: Possible correlations and precise applications in modulating color evolution and orthonasal perception of wines. Food Res. Int..

[51] Guo Z., Dong H., Lin J., Hu Y., Ren D., Yi L., Li S. (2024). Mannoproteins modulate olfactory perception and copigmentation of organoleptic-active-components in wines: Effects and potential molecular mechanisms. Food Res. Int..

[52] Chalier P., Angot B., Delteil D., Doco T., Gunata Z. (2007). Interactions between aroma compounds and whole mannoprotein isolated from *Saccharomyces cerevisiae* strains. Food Chem..

[53] Ginsburg I., Koren E., Shalish M., Kanner J., Kohen R. (2012). Saliva increases the availability of lipophilic polyphenols as antioxidants and enhances their retention in the oral cavity. Arch. Oral Biol..

[54] Li Y., Gao Z., Guo J., Wang J., Yang X. (2022). Modulating aroma release of flavour oil emulsion based on mucoadhesive property of tannic acid. Food Chem..

[55] Manjón E., Brás N.F., García-Estévez I., Escribano-Bailón M.T. (2020). Cell Wall Mannoproteins from Yeast Affect Salivary Protein-Flavanol Interactions through Different Molecular Mechanisms. J. Agric. Food Chem..

[56] Manjón E., Recio-Torrado A., Ramos-Pineda A.M., García-Estévez I., Escribano-Bailón M.T. (2021). Effect of different yeast mannoproteins on the interaction between wine flavanols and salivary proteins. Food Res. Int..

[57] Ramos-Pineda A.M., Manjón E., Macías R.I.R., García-Estévez I., Escribano-Bailón M.T. (2022). Role of Yeast Mannoproteins in the Interaction between Salivary Proteins and Flavan-3-ols in a Cell-Based Model of the Oral Epithelium. J. Agric. Food Chem..

[58] Velázquez-Martínez R.I., Criado C., Muñoz-González C., Crespo J., Pozo-Bayón M.Á. (2023). Evaluation of the Long-Lasting Flavour Perception after the Consumption of Wines Treated with Different Types of Oenological Additives Considering Individual 6-n-Propylthiouracil Taster Status. Foods.

[59] Pérez-Jiménez M., Rocha-Alcubilla N., Pozo-Bayón M.Á. (2019). Effect of saliva esterase activity on ester solutions and possible consequences for the in-mouth ester release during wine intake. J. Texture Stud..

[60] Buettner A. (2004). Investigation of Potent Odorants and Afterodor Development in Two Chardonnay Wines Using the Buccal Odor Screening System (BOSS). J. Agric. Food Chem..

[61] Esteban-Fernández A., Rocha-Alcubilla N., Muñoz-González C., Moreno-Arribas M.V., Pozo-Bayón M.Á. (2016). Intra-oral adsorption and release of aroma compounds following in-mouth wine exposure. Food Chem..

[62] De-La-Fuente-Blanco A., Sáenz-Navajas M.P., Ferreira V. (2016). On the effects of higher alcohols on red wine aroma. Food Chem..

[63] Criado C., Muñoz-González C., Hernández-Ledesma B., Pozo-Bayón M.Á. (2022). Temporal changes in salivary composition induced by oral exposure to different wine matrices and the relationship with the behaviour of aroma compounds in the mouth. Food Funct..

[64] Bojko B., Pawliszyn J. (2012). The benefits of using solid-phase microextraction as a greener sample preparation technique. Bioanalysis.

[65] Kolb B., Ettre L.S. (2006). Static Headspace-Gas Chromatography: Theory and Practice.

[66] Jeleń H.H., Wieczorek M.N. (2023). Commentary: “Quantitative” vs quantitative Headspace Solid-Phase Microextraction (HS-SPME) in food volatile and flavor compounds analysis. J. Food Compos. Anal..

[67] Jackowetz J.N., Orduña R.M. (2013). Survey of SO_2_ binding carbonyls in 237 red and white table wines. Food Control.

[68] Waterhouse A.L., Sacks G.L., Jeffery D.W. (2024). Understanding Wine Chemistry.

[69] Genovese A., Dimaggio R., Lisanti M.T., Piombino P., Moio L. (2005). Aroma composition of red wines by different extraction methods and Gas Chromatography-SIM/MASS spectrometry analysis. Ann. Chim..

[70] Spillman P.J., Iland P.G., Sefton M.A. (1998). Accumulation of volatile oak compounds in a model wine stored in American and Limousin oak barrels. Aust. J. Grape Wine Res..

[71] Pérez Jiménez M., Muñoz González C., Pozo Bayón M.Á. (2020). Understanding human salivary esterase activity and its variation under wine consumption conditions. RSC Adv..

[72] Pérez-Jiménez M., Muñoz-González C., Pozo-Bayón M.Á. (2021). Specificity of Saliva Esterases by Wine Carboxylic Esters and Inhibition by Wine Phenolic Compounds Under Simulated Oral Conditions. Front. Nutr..

[73] Weng Z.M., Ge G.B., Dou T.Y., Wang P., Liu P.K., Tian X.H., Qiao N., Yu Y., Zou L.W., Zhou Q. (2018). Characterization and structure-activity relationship studies of flavonoids as inhibitors against human carboxylesterase 2. Bioorg. Chem..

[74] Zou L.-W., Jin Q., Wang D.-D., Qian Q.-K., Hao D.-C., Ge G.-B., Yang L. (2017). Carboxylesterase Inhibitors: An Update. Curr. Med. Chem..

[75] Mitropoulou A., Hatzidimitriou E., Paraskevopoulou A. (2011). Aroma release of a model wine solution as influenced by the presence of non-volatile components. Effect of commercial tannin extracts, polysaccharides and artificial saliva. Food Res. Int..

[76] Dinnella C., Recchia A., Vincenzi S., Tuorila H., Monteleone E. (2010). Temporary modification of salivary protein profile and individual responses to repeated phenolic astringent stimuli. Chem. Senses.

[77] Ployon S., Morzel M., Canon F. (2017). The role of saliva in aroma release and perception. Food Chem..

[78] Muñoz-González C., Feron G., Guichard E., Rodríguez-Bencomo J.J., Martín-Álvarez P.J., Moreno-Arribas M.V., Pozo-Bayón M.Á. (2014). Understanding the role of saliva in aroma release from wine by using static and dynamic headspace conditions. J. Agric. Food Chem..

[79] Lyu J., Chen S., Xu Y., Li J., Nie Y., Tang K. (2021). Influence of tannins, human saliva, and the interaction between them on volatility of aroma compounds in a model wine. J. Food Sci..

[80] Lyu J., Wang S., Ma Y., Xu Y., Tang K. (2024). Study on the interaction of tannins and salivary proteins affecting wine aroma volatility: Static HS-SPME and molecular dynamics simulation approaches. Food Res. Int..

[81] Velázquez-Martínez R.I., Muñoz-González C., Marina-Ramírez A., Pozo-Bayón M.Á. (2025). Time-dependent changes in the early salivary proteome after oral stimulation with wine differs by the individual 6-n-propylthiouracil (prop) taster status. Food Funct..

