# Supplementary material for: Effect of Oenological Additives on Oral Aroma Retention During Wine Tasting
Source: Foods. 2026 Mar 10;15(6):975. doi: 10.3390/foods15060975 (PMC13025448; doi:10.3390/foods15060975)
Supplement: Supplementary file 1 [file foods-15-00975-s001.zip › foods-4172771-supplementary.pdf]

Supplementary Materials:

Table S1. Oenological additives and wines prepared for the present study.

Oenological Additives	Wines						
	Chemical Composition			White Wines		Red Wines	
	Total Polysaccharide (% mass)	Protein (% mass)	Total Polyphenols (GAE mg/L)	Wine Type	Concentration of Additive	Wine Type	Concentration of Additive
**No additive (Control)**	--	--	--	CWW	--	CRW	--
**Gallotannin**	--	--	1.4	GTWW	300 mg/L	GTRW	300 mg/L
**Ellagitannin**	--	--	1.36	EWW	700 mg/L	ERW	700 mg/L
**Mannoprotein ***	65	3	--	MWW	1.5 mL/L*	MRW	0.9 mL/L

* Commercially available in liquid format. Polysaccharides were determined by using the phenol sulfuric method. Protein determination was carried out using the Kjeldahl method. The concentration of total polyphenols was calculated using the Folin-Ciocalteu method (expressed in mg/L gallic acid equivalents).

Table S2. Percentages of aroma retained in the oral cavity of the volunteers (n = 38) after rinsing with the different types of red and white wines used in this study.

% RETENTION OF AROMA COMPOUNDS

**Wine**	**Wine Type**	**Eugenol**	**Trans-Whiskylactone**	**Cis-Whiskylactone**	**Guaiacol**	**Vanillin**	**Furaneol**	**β-Damascenone**	**Ethyl Cinnamate**	**Isoamyl Acetate**
**Red**	**CRW**	64.65	46.38	41.12	54.15	34.42	58.07	38.45	55.13	50.43
	**GTRW**	73.19	53.74	49.84	69.05	55.11	51.51	50.51	79.69	55.21
	**MRW**	80.01	39.85	40.87	54.87	39.99	57.5	37.1	56.78	66.44
	**ERW**	85.35	54.11	49.44	69.74	58,95	53.86	50.31	76.55	65.71
**White**	**CWW**	87.7	52.5	59.4	86.4	n.d.	n.d.	67.5	83.6	80.8
	**GTWW**	66.6	59.9	59.8	100	n.d.	n.d.	58.4	69.2	58.9
	**MWW**	81.2	93.7	91.1	77.2	n.d.	n.d.	92.3	78.8	85.2
	**EWW**	67.3	46.7	49.0	63.6	n.d.	n.d.	54.6	75.4	56.3

n.d.: not detected aroma compound.

Table S3. Spearman Correlation analysis between percentage of oral aroma retention and physicochemical aroma characteristics for the different wines

**Red Wines**	**Log P**	**BP (°C)**	**MW (g/mol)**
**%AR CRW**	-0.042	0.259	-0.360
**%AR GTRW**	0.192	**0.711**	-0.494
**%AR EWW**	0.159	**0.695**	-0.377
**%AR MRW**	-0.050	0.368	-0.351

**White Wines**	**Log P**	**BP (°C)**	**MW (g/mol)**
**%AR CWW**	0.018	**0.883**	-0.234
**%AR GTWW**	-0.523	0.595	-0.631
**%AR EWW**	0.270	**0.991**	-0.450
**%AR MWW**	0.180	**-0.829**	0.649

Results in bold are statistically significant ($p < 0.05$). %AR: Percentage of oral aroma retention.
